# CMR Mapping: The 4th-Era Revolution in Cardiac Imaging

**DOI:** 10.3390/jcm13020337

**Published:** 2024-01-07

**Authors:** Nazario Carrabba, Mattia Alexis Amico, Andrea Igoren Guaricci, Maria Cristina Carella, Viviana Maestrini, Sara Monosilio, Patrizia Pedrotti, Fabrizio Ricci, Lorenzo Monti, Stefano Figliozzi, Camilla Torlasco, Andrea Barison, Andrea Baggiano, Alessandra Scatteia, Gianluca Pontone, Santo Dellegrottaglie

**Affiliations:** 1 Cardio-Thoraco-Vascular Department, Careggi Hospital, 50134 Florence, Italy; mattiaalexis.amico@unifi.it; 2 University Cardiology Unit, Interdisciplinary Department of Medicine, University of Bari Aldo Moro, 70121 Bari, Italy; 3 Department of Clinical, Anestesiological and Cardiovascular Science, Sapienza University of Rome, 00185 Rome, Italysara.monosilio@uniroma1.it (S.M.); 4 Institute of Sports Medicine and Science of Rome, Comitato Olimpico Nazionale Italiano (CONI), 00197 Rome, Italy; 5 S.S. Cardiologia Diagnostica per Immagini—RM Cardiaca; S.C. Cardiologia 4 Diagnostica-Riabilitativa Dipartimento CardioToracoVascolare “De Gasperis”, ASST Grande Ospedale Metropolitano Niguarda, 20162 Milano, Italy; 6 Department of Neuroscience, Imaging and Clinical Sciences, G. d’Annunzio University of Chieti-Pescara, 66100 Chieti, Italy; 7 IRCCS Humanitas Research Hospital, Via Manzoni 56, Rozzano, 20089 Milan, Italy; lorenzo.monti@humanitas.it (L.M.);; 8 Department of Cardiovascular, Neural and Metabolic Sciences, IRCCS Istituto Auxologico Italiano, 20165 Milan, Italy; c.torlasco@auxologico.it; 9 Fondazione Toscana Gabriele Monasterio, 56127 Pisa, Italy; 10 Department of Perioperative Cardiology and Cardiovascular Imaging, Centro Cardiologico Monzino IRCCS, 20138 Milan, Italy; andrea.baggiano@cardiologicomonzino.it (A.B.); gianluca.pontone@cardiologicomonzino.it (G.P.); 11 Department of Biomedical, Surgical and Dental Sciences, University of Milan, 20133 Milan, Italy; 12 Cardiovascular MRI Laboratory, Unit of Advanced Cardiovascular Imaging, Ospedale Medico-Chirurgico Accreditato Villa dei Fiori, 80011 Acerra, Italy

**Keywords:** cardiac magnetic resonance, parametric mapping, T1 mapping, T2 mapping, ischemic heart disease, hypertrophic cardiomyopathy, dilated cardiomyopathy, inflammatory cardiomyopathies, aortic valve stenosis, athlete’s heart

## Abstract

Cardiac magnetic resonance (CMR) imaging has witnessed substantial progress with the advent of parametric mapping techniques, most notably T1 and T2 mapping. These advanced techniques provide valuable insights into a wide range of cardiac conditions, including ischemic heart disease, cardiomyopathies, inflammatory cardiomyopathies, heart valve disease, and athlete’s heart. Mapping could be the first sign of myocardial injury and oftentimes precedes symptoms, changes in ejection fraction, and irreversible myocardial remodeling. The ability of parametric mapping to offer a quantitative assessment of myocardial tissue properties addresses the limitations of conventional CMR methods, which often rely on qualitative or semiquantitative data. However, challenges persist, especially in terms of standardization and reference value establishment, hindering the wider clinical adoption of parametric mapping. Future developments should prioritize the standardization of techniques to enhance their clinical applicability, ultimately optimizing patient care pathways and outcomes. In this review, we endeavor to provide insights into the potential contributions of CMR mapping techniques in enhancing the diagnostic processes across a range of cardiac conditions.

## 1. Introduction 

Advanced and innovative cardiac magnetic resonance (CMR) techniques, including T1 and T2 mapping, have recently gained considerable attention in the field of cardiac imaging. While they represent notable advancements, their effect on clinical practice remains a subject of investigation. In this review, we explore specific conditions where CMR mapping exhibits its utmost utility, shedding light on its pivotal role in evolving cardiac imaging diagnosis to personalize treatment approaches.

## 2. Parametric Mapping

In the current era, CMR represents the gold-standard imaging technique for assessing cardiac function and accurately measuring ventricular volumes and mass [1]. CMR techniques T1-weighted (T1w) fast-spin echo (FSE) with/without fat saturation, T2-weighted (T2w) short tau inversion recovery (STIR) pulse sequence, and late gadolinium enhancement (LGE) are effective in detecting fat infiltration/metaplasia, edema, and fibrosis, respectively. However, a notable limitation of these conventional techniques is their qualitative or semiquantitative nature, allowing only a comparative analysis between normal and diseased myocardium. This limitation is especially evident in LGE and T2-STIR sequences. 

Parametric mapping can overcome technical challenges encountered in conventional T1w or T2w imaging, including artifacts resulting from extended acquisition times. Additionally, providing a quantitative evaluation of the tissue, mapping plays an important role in cases of diffuse conditions, such as diffuse fibrosis or widespread edema, where a comparative assessment between the pathological area and a reference area may not be feasible due to the absence of virtually normal myocardium. The quantification of myocardial tissue also serves to monitor treatment responses and potentially detect early-stage diseases. This is now evident using artificial intelligence in CMR [2]. Specifically, parametric heart mapping involves the measurement of myocardial relaxation times (T1, T2, and T2*) in every pixel of the acquired image. These relaxation times, expressed in milliseconds, represent distinctive tissue properties, and can vary depending on the CMR field strength. T1 mapping represents the longitudinal relaxation time of the myocardium. In native myocardium, the relaxation time is prolonged when there is an increase in free water content, such as in cases of edema, or when large molecules occupy the interstitium, as observed in conditions involving diffuse fibrosis or amyloid deposition. Vice versa, T1 relaxation times decrease when there is an accumulation of iron within cells or in the extracellular space, as seen in primary or secondary hemochromatosis, or in cases of intracellular lipid accumulation, as in Anderson–Fabry’s disease. T1-mapping images can also be acquired after contrast injection to calculate the myocardial extracellular volume fraction (ECV), which increases in the presence of myocardial edema, diffuse fibrosis, or amyloid deposition. T2 mapping represents the transverse relaxation time of the myocardium. Its elevation is specific to increased myocardial water content, so it is used to accurately assess the presence of myocardial edema. T2* represents transverse relaxation in the presence of magnetic field inhomogeneities. It is shortened in the presence of iron, making it a valuable tool for evaluating myocardial iron overload, such as in primary and secondary hemochromatosis. Additionally, T2* is utilized to detect myocardial hemorrhage, which can occur as a complication of myocardial infarction. The main challenge of CMR mapping development is the lack of standardized protocols on T1 and T2 mapping reference sequences, even leading to vendor-specific variations. Additionally, the establishment of a local normal range is required according to current consensus, which is time-consuming for MRI centers.

**Figure 1:**
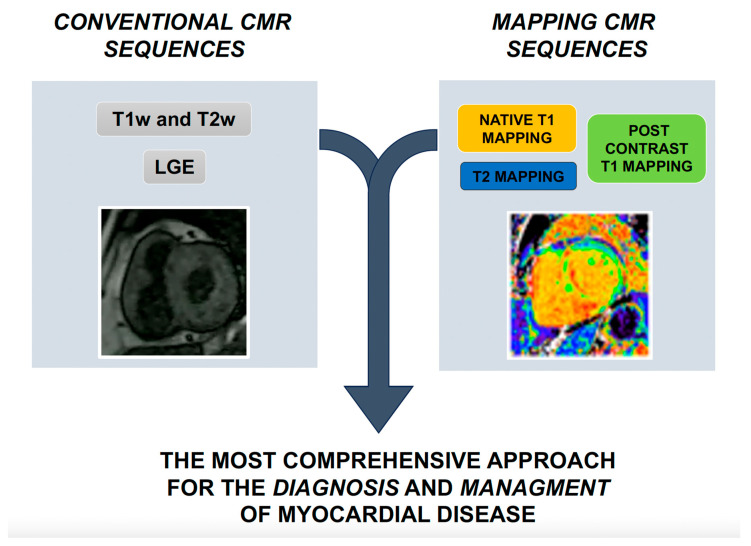
Central Illustration. Integration of novel CMR techniques with conventional sequences. CMR: cardiac magnetic resonance. T1w: T1 weighted. T2w: T2 weighted. LGE: late gadolinium enhancement.

The integration of these novel techniques in current scenarios are summarized in Figure 1 (Central Illustration). In Table 1, we reported the usefulness of traditional and novel CMR pulse sequences in the study of major heart conditions. 

## 3. CMR Mapping and Ischemic Heart Disease

The utility of CMR in ischemic heart disease (IHD) is emphasized in the most recent European and US guidelines due to its capacity to assess the entire pathophysiological pathway of the ischemic cascade [3]. It allows for both functional and morphological characterization of the ischemic heart. On one hand, CMR has rapidly become the gold standard for the functional assessment of the heart, providing an optimal evaluation of myocardial kinesis and wall thickening/thinning, and enabling accurate assessments of ventricular volumes and systolic function. On the other hand, the morphological characterization of the heart is crucial in IHD, relying on diagnostic CMR’s capability to detect myocardial edema and ischemic scarring. In addition, the prognostic role of detecting ischemic scarring via CMR is growing, and it may help us to reclassify patients who really need prophylactic implantable-cardioverter defibrillator therapy [4].

T1w and T2w sequences are routinely used for evaluating cardiac morphology/structure and myocardial edema, respectively. Despite their widespread use and widely accepted diagnostic and prognostic value, in both T1w and T2w sequences the signal intensity is represented on an arbitrary scale and cannot be compared among subjects or across serial testing in a single subject. Thus, these sequences allow only for a semiquantitative approach, requiring a normal tissue as a reference (the “remote myocardium”) for the signal quantification of a region of interest, limiting their importance in cases of diffuse disease. 

Recognition of edema in myocardial diffuse diseases with CMR remains a major challenge. The quantification of global edema can be evaluated through normalizing the signal to skeletal muscle [5,6]. This approach, optimally obtained using a body coil, provides a signal intensity ratio (T2 SI ratio) and may overcome an important limitation of T2w imaging, i.e., artifacts leading to an artificially low signal intensity of the tissue. The T2 SI ratio does not use low-signal-intensity areas as reference regions and thus is not sensitive to such artifacts. On the other hand, the selection of the skeletal muscle is under the discretion of the reader and thus is often a source of observer bias. Furthermore, different skeletal muscles may be more or less suitable as a reference [7]. In addition, artifacts due to the long acquisition times required can also be limiting. Overall, these challenges can result in insufficient diagnostic value in over 20% of cases [8].

The characterization of necrotic tissue and scars is entrusted to LGE acquisition protocols. The LGE phenomenon is caused by delayed gadolinium washout from the diseased versus healthy myocardium, leading to shortening of the T1 times. As a result, areas with focal fibrosis are notably enhanced, appearing brighter on T1w images. LGE imaging also offers insights into the irreversible damage of the microvascular circulation through visualizing microvascular obstruction (MVO) and intramyocardial hemorrhage (IMH). MVO and IMH can be identified as low or absent signal areas in LGE images, typically located within the central portions of the infarcted tissue. However, even for LGE techniques, there are multiple limitations in the current application: small subendocardial enhancements can sometimes be missed, and quantifying diffuse myocardial involvement (e.g., microscopic fibrosis) is challenging. Furthermore, LGE is sensitive to both motion artifacts and incomplete nulling of the myocardium, and it does not differentiate well between acute and chronic myocardial infarction. Moreover, exact quantification of MVO and IMH is challenging due to the spatial definition of these areas and the need for manual planimetry quantification.

To overcome the limitations of traditional qualitative or semiquantitative measurements in evaluating IHD based on T1w and T2w images, parametric mapping has emerged as a promising tool for myocardial characterization. It can provide additional information through advanced quantification of imaging biomarkers [9]. Firstly, through measuring intrinsic tissue properties, mapping allows for a pixel-wise fitting of each decay curve, enabling direct visualization of tissue MR properties, such as T1, T2, and T2*. Characterizing the myocardium using absolute values (e.g., in milliseconds) eliminates the need for a reference to normal remote tissue. This is a significant advantage in cases of diffuse disease presentation, such as diffuse fibrosis, and in scenarios with large infarcts, where the necrotic area might occasionally be underestimated. The direct quantitative comparison of maps within individuals over time can also aid in differentiating between acute and chronic myocardial infarctions and ischemia-reperfusion injuries [10].

T2 mapping quantitatively evaluates tissue water content, and its sensitivity and specificity for myocardial edema have been well-documented [11]. In fact, both in ST-segment elevation myocardial infarction (STEMI) and non-ST-segment elevation myocardial infarction (NSTEMI), baseline T2 myocardial time is significantly elevated in the infarct zone, decreasing to near-normal values six months after reperfusion. This differentiation of the timing of infarction is achieved with higher diagnostic accuracy than semiquantitative T2w methods [12]. T2 mapping also outperforms T2w imaging in identifying infarct-related arteries and estimating the area of myocardial injury in NSTEMI [13]. Furthermore, both T2 and T1 mapping correlate well with the area at risk (AAR), which is the surrounding zone of a necrotic area where cells can still recover if coronary reperfusion is rapidly established [14]. T2 mapping is also valuable for detecting MVO and IMH, underscoring its role in prognostication after reperfusion therapy [15], even though T2* mapping remains the standard for this quantification. T1 mapping can assess the transmural extent of myocardial infarction thereby differentiating viable from non-viable myocardium without the use of LGE in both acute and chronic myocardial infarction. Moreover, T1 mapping performed better in chronic compared to acute myocardial infarction due to the absence of myocardial edema [16].

Another significant implication is the ability of T1 mapping techniques to visualize permanent myocardial injury [17]. The advantage of performing mapping without the need for contrast agents makes the exam accessible and safe for individuals with chronic kidney disease and allows for multiple acquisitions over time, facilitating patient follow-up. Furthermore, it offers a quantitative method independent of the need for a reference myocardium for comparison, eliminating the manual contouring and semi-automated approaches often used for LGE evaluation. T1 mapping also enables the assessment of forms of diffuse fibrosis that may be missed by conventional approaches [18]. Additionally, T1-mapping protocols allow for the calculation of ECV and the generation of ECV maps, estimated through measuring pre- and post-contrast relaxivity changes adjusted via hematocrit, serving as a surrogate for diffuse myocardial fibrosis.

## 4. CMR Mapping and Cardiomyopathies

Cardiomyopathies constitute a heterogeneous group of myocardial disorders, demanding a comprehensive approach to their diagnosis. As emphasized by the latest ESC guidelines [19], CMR plays a pivotal role in the non-invasive assessment of cardiomyopathies, offering unparalleled insights into their pathophysiology and facilitating early diagnoses and personalized treatment strategies. In particular, CMR parametric mapping can visualize any change in myocardial composition spacing, from evaluation of cardiomyocyte intracellular deposits (e.g., iron overload in hemochromatosis, or glycosphingolipid in Anderson–Fabry disease) to extracellular modifications of the interstitium (e.g., myocardial fibrosis or accumulation of collagen or amyloid proteins) [20]. This multifaceted approach provides invaluable insights into the pathological processes at play, offering a level of detail that was previously attainable only through invasive histological examinations. 

**Figure 2:**
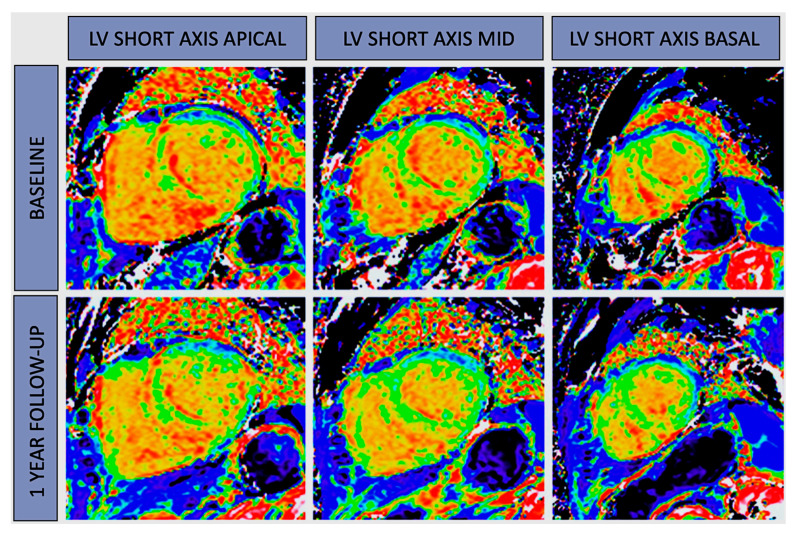
ECV mapping in amyloidosis. CMR-derived ECV map at baseline and after 12 months of Tafamidis treatment in a patient with amyloidosis (52.1 ± 8.2 vs. 49 ± 8.5, %). Tafamidis stabilized cardiac amyloidosis deposition in hereditary transthyretin cardiomiopathy. LV: left ventricular.

In the realm of hypertrophic cardiomyopathy (HCM) research, cardiac MRI stands as a valuable tool for comprehensively characterizing the heart. It’s widely acknowledged for its ability to assess critical parameters, such as myocardial thickness, extracellular volume (ECV), and regional strain, all of which play pivotal roles in understanding this condition. Notably, T2 mapping has emerged as an essential component in assessing the severity of hypertrophy. In fact, T2 time variations are more pronounced in relation to hypertrophy severity compared to T1 time prolongation. The utilization of LGE to pinpoint areas of myocardial fibrosis is not only diagnostically valuable but also holds significant prognostic implications. However, the introduction of T1 mapping technology has helped mitigate some of the traditional challenges associated with LGE quantification. Elevated native T1 values now allow us to identify diffuse fibrosis or areas of interest even when LGE findings are absent [9,10]. Understanding myocardial edema, represented by T2 prolongation, is a complex task influenced by factors like collagen accumulation, ischemia, and microvascular dysfunction [21]. T2 mapping, therefore, emerges as a valuable tool, particularly in distinguishing HCM from physiological left ventricular hypertrophy (LVH) in athletes. Elevated T1 and ECV measurements show strong associations with the left ventricular mass index across the entire spectrum of HCM patients, aiding in distinguishing various phenocopies. For example, consider Anderson–Fabry disease (FD) where a notably reduced native T1 value serves as a red flag, raising suspicions of FD. This reduction is particularly prominent at the basal septum and often precedes ventricular hypertrophy. However, it’s crucial to acknowledge that at more advanced stages, inflammation and lymphocyte recall can lead to a pseudo-normalization of T1 time, potentially misleading clinicians. Experts have proposed a three-phase model for FD, encompassing accumulation, inflammatory, and terminal phases [22]. On the other hand, in the evaluation of cardiac amyloidosis (CA), the accumulation of amyloid fibrils triggers an expansion in ECV, resulting in myocardial damage and edema. The LGE patterns in CA patients exhibit a characteristic evolution, transitioning from a fuzzy and focal appearance in earlier stages to diffuse, subendocardial, transmural, or binary patterns in advanced cases. Native T1 mapping has now emerged as a sensitive and specific diagnostic tool for identifying light-chain (AL) and ATTR CA without necessitating contrast agents. In light-chain amyloid cardiomyopathy, the combined influence of amyloid accumulation (ECV) and myocardial edema (T2) is reflected in native T1 values [23]. In addition, serial ECV mapping may lead us to evaluate the treatment efficacy for amyloid cardiomyopathy (Figure 2).

The role of cardiac mapping in arrhythmogenic right ventricular cardiomyopathy (ARVC) is a subject of ongoing debate. Cardiac MRI plays a crucial role in assessing structural abnormalities of the right ventricle, which is essential for diagnosing ARVC. This is particularly important as the accuracy of transthoracic echocardiography in defining right ventricular (RV) structure and function is often inadequate. Cardiac MRI mapping is not only useful for diagnosis and risk stratification of ARVC, identifying regions of fibrofatty replacement in the right or left ventricle, but it also helps detect early-stage disease and guide patient management. In this context, T1 mapping becomes an essential tool for clinicians. Native T1 values are notably elevated in early-stage patients and individuals at risk within affected families. There are even anecdotal cases demonstrating how parametric CMR mapping assists in the non-invasive diagnosis of arrhythmogenic cardiomyopathy, even when it involves the left ventricle [24].

Dilated cardiomyopathy (DCM) necessitates the use of cardiac MRI mapping for a comprehensive assessment of myocardial viability, fibrosis distribution, and contractility. Anomalies in native myocardial T1 relaxation times emerged as potential indicators of an unfavorable prognosis among individuals with DCM. Additionally, extracellular volume fraction (ECV) exhibited a robust correlation with major adverse cardiac events (MACE) across all anatomical regions, with the strongest association identified in the anteroseptal region. Quantitative CMR features for MACEs in DCM patients showed potential predictive value. In the context of a chronic presentation of DCM, myocardial T2 relaxation time is notably prolonged, regardless of the extent of left ventricular dysfunction [25]. T2 mapping enhances the identification of early-stage DCM, especially when myocardial morphology is challenging to differentiate from athletic myocardial adaptation. Although the burden of non-ischemic scarring detected via LGE is a useful tool for indication of implantable cardioverter defibrillators (ICDs) [26], the incorporation of quantitative CMR markers allows for a tailored approach to therapeutic strategies, including the placement of implantable cardioverter defibrillators (ICDs) and cardiac resynchronization therapy.

MRI is used to evaluate restrictive cardiomyopathy (RCM), but the use of tissue characterization parameters is not widely documented. These parameters could help distinguish RCM from constrictive pericarditis (CP) and other forms of cardiomyopathy. T1w sequences and cine-SSFP imaging can reveal widespread thickening of pericardial layers accompanied by hypointense calcifications in CP patients, despite nearly 20% exhibiting normal pericardial thickness. T1 and T2 mapping techniques can identify conditions of accumulation or infiltration associated with altered myocardial relaxation properties or other restrictive conditions causing diffuse inflammation or fibrosis [27]. One of the most important uses of MRI mapping in RCM is to identify conditions leading to iron overload in the myocardium. T2* mapping is a highly specific method that allows quantitative assessment of iron levels in the heart and liver. However, native T1 mapping has the potential to improve the detection of mild iron burden, but this superior reproducibility is lost in the presence of significant iron accumulation. ECV does not currently play a role in the management of patients with cardiac siderosis.

## 5. CMR Mapping and Myocarditis 

The Lake Louise Criteria (LLC) have been widely utilized for the diagnosis of myocarditis through CMR [28]. They establish a diagnosis of myocardial inflammation when a minimum of two out of the following three tissue-based CMR markers are observed:

(a) Edema: this is detected as high signal intensity in the myocardium on STIR T2w images.
(b) Hyperemia: This is characterized by increased regional gadolinium contrast agent uptake in the abnormal myocardium during the initial minutes following the injection, commonly referred to as early gadolinium enhancement (EGE). While EGE was initially part of the LLC criteria, later studies demonstrated that its exclusion from the original criteria does not seem to significantly affect their diagnostic accuracy [29]. Alternatively, hyperemia can be assessed using traditional cine steady-state free precession images acquired shortly after contrast administration.
(c) Fibrosis/Necrosis: this is depicted as myocardial contrast deposition with subepicardial/intramyocardial distribution on LGE images.

The introduction of parametric mapping images has addressed the traditional limitations of T1w or T2w images and has gained significant recognition in the updated 2018 LLC [30]. In this revised version of the criteria, the confirmation of acute myocardial inflammation involves the presence of at least one CMR marker for edema, observed on either T2w images or T2 mapping, in conjunction with the presence of at least one T1-based marker indicative of associated myocardial injury. These markers for myocardial injury can be assessed on LGE images, T1 mapping, or through extracellular volume fraction measurements. The incorporation of mapping techniques has demonstrated a substantial improvement in the diagnostic accuracy of CMR for myocarditis [31]. Furthermore, parametric mapping techniques offer a valuable alternative for detecting myocardial inflammation using CMR in situations where the administration of contrast agents must be avoided.

Elevated native T1 values are indicative of myocardial inflammation, likely arising from a complex interplay of factors including intracellular and extracellular myocardial edema, hyperemia, capillary leakage, and myocyte necrosis. In the context of conditions like viral myocarditis, rheumatoid arthritis, and systemic lupus erythematosus, native T1 values have been found to increase. However, it’s important to note that T1 values can also rise in regions with myocardial fibrosis, attributed to the expansion of the extracellular space or myocardial damage. Consequently, interpreting these findings becomes challenging, as it may be unclear whether they reflect active inflammation, chronic fibrosis, or a combination of both. Furthermore, T2 mapping offers distinct advantages over conventional T2w imaging in the detection of acute myocardial inflammation and edema. It excels in differentiating both focal and global myocardial edema. Additionally, T2 mapping effectively addresses many common limitations associated with T2-STIR, such as incomplete blood suppression, signal dropouts in the lateral wall, and lower signal-to-noise ratios. Finally, in patients with chronic myocarditis, only T2 mapping has acceptable diagnostic performance [32], and is useful for risk stratification [33]. The degree of T2 relaxation time prolongation, and to a lesser extent the percentage of myocardium with prolonged T2 time, are reliable predictors of MACE and heart failure (HF) hospitalization in patients with myocarditis. T2 time tends to shorten after resolution of the inflammation, whereas LGE may persist. Persistent time prolongation after the acute phase correlates with MACE, HF hospitalization, and LV dysfunction, making T2 mapping a useful monitoring tool in patients with myocarditis [33].

## 6. CMR Mapping and Inflammatory Cardiomyopathies

Inflammatory cardiomyopathies represent a heterogeneous group of heart muscle disorders characterized by non-ischemic inflammation of the myocardium that can involve the myocardium as a response to a wide range of etiologies, such as infectious agents, drugs, and toxins, or systemic immune-mediated diseases [34]. These conditions can be challenging to diagnose and manage due to their varied etiologies and, more importantly, their different clinical presentations. CMR represents the gold-standard imaging test in cases of suspected inflammatory cardiomyopathy in both acute and chronic settings [35]. Furthermore, CMR shows remarkable diagnostic inter-observer agreement as well as an optimal safety profile, and holds potential to facilitate patient monitoring, assess therapeutic efficacy, and provide prognostic information [36].

In inflammatory cardiomyopathy, ECV is influenced by several factors such as acute extracellular edema or interstitial changes following myocyte necrosis, focal replacement fibrosis, or diffuse myocardial fibrosis. ECV results elevated both in evident biopsy-proven myocarditis as well as in subclinical forms of myocardial involvement, shedding light on subtle manifestations of myocardial involvement in conditions like rheumatoid arthritis, systemic lupus erythematosus, and systemic sclerosis. These observations highlight the diagnostic potential of ECV in cases involving low-grade inflammation and diffuse fibrosis, aspects that may be challenging to detect using traditional CMR techniques [34].

T1 and T2 mapping techniques emerged as game-changing technology in the diagnosis, prognosis, and management of inflammatory cardiomyopathies. To underscore the significance of these innovative methods, a study conducted by Cundari et al. [37] emphasized that CMR’s diagnostic accuracy can be significantly enhanced when T1 and T2 mapping are employed, especially in challenging cases. Interestingly, in patients with an “infarct-like” presentation, both the revised and the old LLC perform well; on the contrary, when myocarditis presents atypically with symptoms such as heart failure or arrhythmias, the revised LLC outperform the old criteria. This improved performance is attributed to the incorporation of parametric techniques capable of detecting subtle alterations in myocardial tissue, allowing for more accurate diagnosis in these scenarios.

## 7. CMR Mapping and Aortic Valve Stenosis 

Valve aortic stenosis, one of the most common valvular diseases in the Western world, is characterized by a progressive left ventricular remodeling response in which myocardial fibrosis and cell death are considered important drivers in this pathological progress [38,39]. The role of CMR in this scenario is useful since it can identify focal and diffuse fibrosis, which are associated with a poor long-term prognosis. Although the remodeling ends after aortic valve replacement (AVR), fibrosis appears irreversible and leads to a persistent, poor long-term prognosis. LGE is reliable, well-validated, and easily integrated into the standard workflow, but it is insensitive to the detection of diffuse interstitial fibrosis that is, on the contrary, easily detected via T1 mapping [40]. Native T1 values increase with fibrosis, reflecting the state of both the intracellular and extracellular environments, while the addition of gadolinium-based contrast agents (post-contrast T1 mapping) facilitates interrogation of the extracellular space. Native T1 has been utilized in several cardiac pathologies demonstrating significant prognostic power and data are also emerging for native T1 in aortic stenosis, although there are still no universal cutoffs for this disease. Post-contrast T1-mapping techniques allow more specific interrogation of the extracellular space. Unfortunately, standardization of these values is difficult due to variations in gadolinium kinetics between patients and even within the same individual on different days. Extracellular volume fraction (ECV%) introduces a way to correct post-contrast myocardial T1-mapping values for blood concentrations of gadolinium agent. 

A crucial feature in various cardiac conditions, including aortic stenosis, is the accumulation of excessive collagen in the interstitial space and the consequent expansion of the extracellular compartment. In this context, the assessment of ECV emerges as a valuable method for detecting diffuse myocardial fibrosis [41]. However, it’s important to note that data on the prognostic value of ECV in aortic stenosis are somewhat limited. In addition to ECV, which offers only a percentage estimate of fibrosis burden, a novel parameter called indexed extracellular volume (iECV) has been introduced. The iECV parameter quantifies the entire left ventricular myocardial volume in relation to the patient’s body surface area. This innovative approach provides a more effective means of assessing the extent of myocardial fibrosis and demonstrates superior discriminative power across various disease states compared to other T1-mapping parameters [42]. Both iECV and ECV have been employed to explore changes in intracellular and extracellular compartments before and after AVR, aiding in understanding how the left ventricle remodels in response to the intervention. Before AVR, there is a balanced increase in both the extracellular matrix and left ventricular mass, which keeps the ECV stable. After AVR, there is a more rapid reduction in cellular mass compared to extracellular mass, resulting in an apparent increase in ECV percentage. However, iECV decreases as it represents the total extracellular matrix volume, not just a percentage. This suggests a potential reversal of diffuse fibrosis [43,44], in contrast to the irreversible nature of replacement fibrosis as assessed via LGE. While iECV requires further validation, it holds promise as a method for monitoring changes in myocardial fibrosis and understanding the effects of treatment interventions.

T1 mapping represents an exciting and evolving field in aortic stenosis research, offering a distinct approach to detect reversible diffuse myocardial fibrosis. Nevertheless, additional research is imperative to establish validated thresholds that can guide clinical decision making. Within the realm of T1 mapping, ECV and iECV emerge as techniques that provide comprehensive insights into cellular and extracellular remodeling in aortic stenosis. However, native T1 mapping has its advantages, including ease of calculation and the avoidance of contrast administration. CMR assessments aimed at detecting diffuse fibrosis in aortic stenosis demand further validation but hold the potential to identify early stages of myocardial disease.

## 8. CMR Mapping and Arrhythmic Mitral Valve Prolapse

The genesis of arrhythmias in individuals with mitral valve prolapse involves an intricate interplay of various factors that remain incompletely understood. Cardiac imaging plays a pivotal role in identifying high-risk features that may potentially elevate the risk of arrhythmic complications [40]. Echocardiographic indicators categorized as “high-risk” encompass the presence of a bileaflet mitral valve prolapse (MVP) [41], coupled with morphofunctional abnormalities of the mitral annulus, such as mitral annular disjunction (MAD) and systolic curling [42]. Another critical aspect characterizing “arrhythmic mitral valve prolapse” is the existence of fibrosis, conventionally identified in late gadolinium enhancement (LGE) sequences in the inferior wall, inferolateral wall, and papillary muscles, as elucidated in the seminal work of Basso C et al. [43].

Costant Dit Beaufils et al. demonstrated that the prevalence of myocardial fibrosis was 13% in trace-mild mitral regurgitation (MR), 28% in moderate MR, and 37% in severe MR. This fibrosis was correlated with specific mitral valve apparatus features, a more dilated left ventricle (LV), and a higher frequency of ventricular arrhythmias (45% vs. 26%, *p* < 0.0001) [44]. The research underscores how the presence of fibrosis, combined with alterations in the mitral valve apparatus, is associated with a more dilated LV, a higher degree of MR, and ventricular arrhythmias—factors independently contributing to the risk of adverse cardiovascular events.

Beyond the localized fibrotic areas identified through LGE, the evaluation of interstitial fibrosis using native T1 mapping or extracellular volume (ECV) has recently been explored for enhanced arrhythmic risk stratification [45,46,47]. Within this context, individuals with MVP exhibited a heightened degree of ventricular remodeling, extending beyond the scope of detection in LGE sequences, as evidenced through elevated native T1 relaxation time or increased ECV. Additionally, an ECV exceeding 33% was identified as having equivalent predictive value for out-of-hospital cardiac arrest as LGE [45].

## 9. CMR Mapping and Athlete’s Heart 

Athlete’s heart is a form of adaptive cardiac remodeling influenced by regular and prolonged exercise that could be so pronounced to generate difficulties in differential diagnosis with early stages of cardiomyopathies [45]. The importance of the differential diagnosis from hypertrophic and arrhythmogenic cardiomyopathies is crucial, so the use of CMR for athletic populations is growing [46]. 

Over the last decade, mapping techniques have experienced a huge expansion in several cardiac conditions, but data on parametric mapping among the athletic population are currently limited. The first studies exploring tissue characterization in a small cohort of athletes showed similar LV mass but lower values of both native T1 and ECV compared to hypertrophic phenocopies, probably due more to cellular hypertrophy rather than extracellular compart expansion in athletes [47]. Nevertheless, Swoboda et al. demonstrated that myocyte hypertrophy regresses after a detraining period [48], corroborating such observations. These findings could have played an important role to differentiate this adaptive cardiac remodeling with HCM or CDM [49,50], but subsequent works on older athletes did not observe significant differences in T1 mapping and ECV between athletes and controls, making even more uncertain the real role of T1 mapping in these subsets [51,52].

Data on T2 mapping are even more scarce. Malek et al. [51] reported higher T2 mapping values in athletes than in controls, which could be primarily due to reversible myocardial injury and edema due to high-intensity exercise [53]. However, a study by Tahir and colleagues who evaluated ultra-marathon runners before and after a race demonstrated the lack of changes in both T1 and T2 mapping values, while concomitant troponin increase after the race was found [54]. Otherwise, when comparing athletes with cardiomyopathy patients rather than sedentary controls, T2 mapping was higher in patients with hypertrophic and dilated cardiomyopathy [50,55]. 

The disparities in the literature may stem from several factors. First, these studies often entail small sample sizes, primarily focusing on endurance athletes. Furthermore, the heterogeneity in study designs adds complexity, as some investigations emphasize distinctions between athletes and sedentary individuals, while others draw comparisons between athletes and those diagnosed with cardiomyopathy. Additionally, certain studies evaluate athletes both before and after high-intensity training, further contributing to variation. Notably, the establishment of mapping reference values proves challenging, particularly within the unique population of athletes. The lack of homogeneity in parametric mapping references and values plays a central role in this complexity. Moreover, within the athletic population, mapping values across different sports categories and genders are notably underrepresented. Hence, there is a compelling need for more extensive studies encompassing diverse athletic groups, thereby facilitating the definition of reference ranges and enhancing our understanding of parametric mapping behavior in this population, which holds significant clinical implications. 

## 10. Conclusions

To date, it is advisable to utilize both conventional and mapping CMR techniques as complementary tools in the assessment of most cardiac diseases. Conventional techniques demonstrate superiority in evaluating myocardial diseases with segmental distribution, such as myocardial infarction, hypertrophic cardiomyopathy, and myocarditis. Conversely, mapping techniques prove more effective in cardiac conditions characterized by diffuse involvement of the myocardium, such as amyloidosis and Fabry disease. However, regional or diffuse involvement of myocardial disease can often be present at the same time or manifest at different times. Therefore, it is reasonable to consider that integrating findings from conventional techniques with mapping features may represent the most comprehensive approach for the diagnosis of myocardial diseases. The main challenge of what is widely regarded as the 4th era of myocardial CMR development is the lack of standardized protocols on T1 and T2 mapping reference sequences, even leading to vendor-specific variations. Additionally, the establishment of a local normal range is required according to current consensus, which is time-consuming for MRI centers. The future advancement of CMR parametric mapping greatly hinges on achieving standardization, which would enable its widespread clinical application. This, in turn, is expected to play a pivotal role in optimizing clinical pathways and ultimately improving patient care.

## Figures and Tables

**Table 1:** Usefulness of CMR traditional and novel pulse sequences in the study of major heart conditions. Each sequence technique is evaluated according to its clinical utility in each cardiac disease with the following grading: − not useful; +/− uncertain usefulness; + useful; ++ very useful. Cine CMR refers to balanced steady-state-free-precession gradient echo sequence, spoiled gradient echo cine sequence with/without contrast agent. Black blood CMR refers to T1-weighted (half-Fourier, single-shot, fast spin echo, double inversion recovery) dark blood spin echo sequence with or without contrast agent; with or without fat saturation, proton-density weighted spin echo sequence. Edema CMR refers to T2-weighted single-shot/fast spin echo double-inversion recovery/triple-inversion recovery dark/black blood spin echo sequence/T2-prepared SSFP with/without fat saturation. Stress perfusion CMR refers to T1-weighted saturation recovery gradient echo sequence with echo-planar, SSFP, or hybrid read-out. LGE CMR refers to 2D/3D inversion-recovery gradient echo sequence, regular/single-shot 2D/3D phase-sensitive SSFP, delayed (hyper) enhancement sequence. Flow CMR: in plane/through-plane motion-encoded phase-sensitive spoiled gradient echo sequence. CMR: cardiac magnetic resonance. T1w: T1-weighted. T2w: T2-weighted. LGE: late gadolinium enhancement. ECV: extracellular volume. T2*: T2-star.

	Cine CMR	Edema CMR	Stress Perfusion CMR	LGE CMR	Native T1 Mapping	ECV Mapping	T2 Mapping	T2* Mapping
Ischemic Heart Disease	++	++	++	++	+	+	++	+/−
Cardiomyopathies	++	+	+/−	++	++	++	++	+
Inflammatory cardiomyopathies	++	++	+/−	++	++	++	++	+/−
Aortic valve disease	++	+/−	+	++	++	++	+/−	-
Arrhythmic mitral valve prolapse	++	+/−	-	++	++	++	++	-
Athlete’s heart	++	+	+/−	++	++	++	+	-

## Data Availability

Not applicable.

## References

[1] Haaf P., Garg P., Messroghli D.R., Broadbent D.A., Greenwood J.P., Plein S. (2016). Cardiac T1 Mapping and Extracellular Volume (ECV) in clinical practice: A comprehensive review. J. Cardiovasc. Magn. Reson..

[2] Ghanbari F., Joyce T., Lorenzoni V., Guaricci A.I., Pavon A.-G., Fusini L., Andreini D., Rabbat M.G., Aquaro G.D., Abete R. (2023). AI Cardiac MRI Scar Analysis Aids Prediction of Major Arrhythmic Events in the Multicenter DERIVATE Registry. Radiology.

[3] Messroghli D.R., Moon J.C., Ferreira V.M., Grosse-Wortmann L., He T., Kellman P., Mascherbauer J., Nezafat R., Salerno M., Schelbert E.B. (2017). Clinical recommendations for cardiovascular magnetic resonance mapping of T1, T2, T2 and extracellular volume: A consensus statement by the Society for Cardiovascular Magnetic Resonance (SCMR) endorsed by the European Association for Cardiovascular Imagin. J. Cardiovasc. Magn. Reson..

[4] Pontone G., Guaricci A.I., Fusini L., Baggiano A., Guglielmo M., Muscogiuri G., Volpe A., Abete R., Aquaro G., Barison A. (2023). Cardiac Magnetic Resonance for Prophylactic Implantable-Cardioverter Defibrillator Therapy in Ischemic Cardiomyopathy: The DERIVATE–ICM International Registry. JACC Cardiovasc. Imaging.

[5] Abdel-Aty H., Boyé P., Zagrosek A., Wassmuth R., Kumar A., Messroghli D., Bock P., Dietz R., Friedrich M.G., Schulz-Menger J. (2005). Diagnostic performance of cardiovascular magnetic resonance in patients with suspected acute myocarditis: Comparison of different approaches. J. Am. Coll. Cardiol..

[6] Röttgen R., Christiani R., Freyhardt P., Gutberlet M., Schultheiss H.P., Hamm B., Kühl U. (2011). Magnetic resonance imaging findings in acute myocarditis and correlation with immunohistological parameters. Eur. Radiol..

[7] Carbone I., Childs H., Aljizeeri A., Merchant N., Friedrich M.G. (2015). Importance of Reference Muscle Selection in Quantitative Signal Intensity Analysis of T2-Weighted Images of Myocardial Edema Using a T2 Ratio Method. Biomed Res. Int..

[8] McCann G.P., Khan J.N., Greenwood J.P., Nazir S., Dalby M., Curzen N., Hetherington S., Kelly D.J., Blackman D.J., Ring A. (2015). Complete Versus Lesion-Only Primary PCI the Randomized Cardiovascular MR CvLPRIT Substudy. J. Am. Coll. Cardiol..

[9] Ferreira V.M., Piechnik S.K. (2020). CMR parametric mapping as a tool for myocardial tissue characterization. Korean Circ. J..

[10] Salerno M., Kramer C.M. (2013). Advances in parametric mapping with CMR imaging. JACC Cardiovasc. Imaging.

[11] O’Brien A.T., Gil K.E., Varghese J., Simonetti O.P., Zareba K.M. (2022). T2 mapping in myocardial disease: A comprehensive review. J. Cardiovasc. Magn. Reson..

[12] Tahir E., Sinn M., Bohnen S., Avanesov M., Säring D., Stehning C., Schnackenburg B., Eulenburg C., Wien J., Radunski U.K. (2017). Acute versus chronic myocardial infarction: Diagnostic accuracy of quantitative Native T1 and T2 mapping versus assessment of edema on Standard T2-weighted cardiovascular MR images for differentiation. Radiology.

[13] Layland J., Rauhalammi S., Lee M.M.Y., Ahmed N., Carberry J., May V.T.Y., Watkins S., McComb C., Mangion K., McClure J.D. (2017). Diagnostic accuracy of 3.0-T magnetic resonance T1 and T2 mapping and T2-weighted dark-blood imaging for the infarct-related coronary artery in Non-ST-segment elevation myocardial infarction. J. Am. Heart Assoc..

[14] Bulluck H., White S.K., Rosmini S., Bhuva A., Treibel T.A., Fontana M., Abdel-Gadir A., Herrey A., Manisty C., Wan S.M.Y. (2015). T1 mapping and T2 mapping at 3T for quantifying the area-at-risk in reperfused STEMI patients. J. Cardiovasc. Magn. Reson..

[15] Carrabba N., Parodi G., Maehara A., Pradella S., Migliorini A., Valenti R., Colagrande S., Mintz G., Antoniucci D. (2013). Effects of rheolytic thrombectomy and manual thrombus aspiration on infarct size and microvascular obstruction during primary angioplasty: Smart–mri substudy. J. Am. Coll. Cardiol..

[16] Dastidar A.G., Harries I., Pontecorboli G., Bruno V.D., Garate E., De Moret C., Baritussio A., Johnson T.W., McAlindon E., Bucciarelli-Ducci C. (2019). Native T1 mapping to detect extent of acute and chronic myocardial infarction: Comparison with late gadolinium enhancement technique. Int. J. Cardiovasc. Imaging.

[17] Schwitter J., Arai A.E. (2011). Assessment of cardiac ischaemia and viability: Role of cardiovascular magnetic resonance. Eur. Heart J..

[18] Weingärtner S., Akçakaya M., Basha T., Kissinger K.V., Goddu B., Berg S., Manning W.J., Nezafat R. (2013). Combined saturation/inversion recovery sequences for improved evaluation of scar and diffuse fibrosis in patients with arrhythmia or heart rate variability. Magn. Reson. Med..

[19] Arbelo E., Protonotarios A., Gimeno J.R., Arbustini E., Barriales-Villa R., Basso C., Bezzina C.R., Biagini E., Blom N.A., de Boer R.A. (2023). 2023 ESC Guidelines for the management of cardiomyopathies: Developed by the task force on the management of cardiomyopathies of the European Society of Cardiology (ESC). Eur. Heart J..

[20] Guglielmo M., Fusini L., Muscogiuri G., Baessato F., Loffreno A., Cavaliere A., Rizzon G., Baggiano A., Rabbat M.G., Muratori M. (2020). T1 mapping and cardiac magnetic resonance feature tracking in mitral valve prolapse. Eur. Radiol..

[21] Huang L., Ran L., Zhao P., Tang D., Han R., Ai T., Xia L., Tao Q. (2019). MRI native T1 and T2 mapping of myocardial segments in hypertrophic cardiomyopathy: Tissue remodeling manifested prior to structure changes. Br. J. Radiol..

[22] Nordin S., Kozor R., Medina-Menacho K., Abdel-Gadir A., Baig S., Sado D.M., Lobascio I., Murphy E., Lachmann R.H., Mehta A. (2019). Proposed Stages of Myocardial Phenotype Development in Fabry Disease. JACC Cardiovasc. Imaging.

[23] Fontana M., Pica S., Reant P., Abdel-Gadir A., Treibel T.A., Banypersad S.M., Maestrini V., Barcella W., Rosmini S., Bulluck H. (2015). Prognostic value of late gadolinium enhancement cardiovascular magnetic resonance in cardiac amyloidosis. Circulation.

[24] Dowd R., Dhanjal T., Schmucki M., Kanagala P., Khan J.N. (2020). Unique role of cardiovascular magnetic resonance imaging parametric mapping in the diagnosis of arrhythmogenic left ventricular cardiomyopathy. Eur. Heart J. Cardiovasc. Imaging.

[25] Spieker M., Katsianos E., Gastl M., Behm P., Horn P., Jacoby C., Schnackenburg B., Reinecke P., Kelm M., Westenfeld R. (2017). T2 mapping cardiovascular magnetic resonance identifies the presence of myocardial inflammation in patients with dilated cardiomyopathy as compared to endomyocardial biopsy. Eur. Heart J. Cardiovasc. Imaging.

[26] Guaricci A.I., Masci P.G., Muscogiuri G., Guglielmo M., Baggiano A., Fusini L., Lorenzoni V., Martini C., Andreini D., Pavon A.G. (2021). CarDiac magnEtic Resonance for prophylactic Implantable-cardioVerter defibrillAtor ThErapy in Non-Ischaemic dilated CardioMyopathy: An international Registry. Europace.

[27] Mitropoulou P., Georgiopoulos G., Figliozzi S., Klettas D., Nicoli F., Masci P.G. (2020). Multi-Modality Imaging in Dilated Cardiomyopathy: With a Focus on the Role of Cardiac Magnetic Resonance. Front. Cardiovasc. Med..

[28] Friedrich M.G., Sechtem U., Schulz-Menger J., Alakija P., Cooper L.T., White J.A., Gutberlet M., Prasad S., Aletras A. (2009). Cardiovascular MRI in myocarditis. J. Am. Coll. Cardiol..

[29] Chu G.C.W., Flewitt J.A., Mikami Y., Vermes E., Friedrich M.G. (2013). Assessment of acute myocarditis by cardiovascular MR: Diagnostic performance of shortened protocols. Int. J. Cardiovasc. Imaging.

[30] Ferreira V.M., Schulz-Menger J., Holmvang G., Kramer C.M., Carbone I., Sechtem U., Kindermann I., Gutberlet M., Cooper L.T., Liu P. (2018). Cardiovascular Magnetic Resonance in Nonischemic Myocardial Inflammation: Expert Recommendations. J. Am. Coll. Cardiol..

[31] Eichhorn C., Greulich S., Bucciarelli-Ducci C., Sznitman R., Kwong R.Y., Gräni C. (2022). Multiparametric Cardiovascular Magnetic Resonance Approach in Diagnosing, Monitoring, and Prognostication of Myocarditis. JACC Cardiovasc. Imaging.

[32] Lurz P., Luecke C., Eitel I., Föhrenbach F., Frank C., Grothoff M., Waha SDe Rommel K.P., Lurz J.A., Klingel K., Kandolf R. (2016). Comprehensive Cardiac Magnetic Resonance Imaging in Patients with Suspected Myocarditis the MyoRacer-Trial. J. Am. Coll. Cardiol..

[33] Spieker M., Aberkorn S., Gastl M., Behm P., Katsianos S., Horn P., Jacoby C., Schnackenburg B., Reinecke P., Kelm M. (2017). Abnormal T2 mapping cardiovascular magnetic resonance correlates with adverse clinical outcome in patients with suspected acute myocarditis. J. Cardiovasc. Magn. Reson..

[34] Thomas K.E., Fotaki A., Botnar R.M. (2023). Europe PMC Funders Group Imaging methods: Magnetic resonance imaging. Circ. Cardiovasc. Imaging.

[35] Tymińska A., Ozierański K., Skwarek A., Kapłon-Cieślicka A., Baritussio A., Grabowski M., Marcolongo R., Caforio A.L.P. (2022). Personalized Management of Myocarditis and Inflammatory Cardiomyopathy in Clinical Practice. J. Pers. Med..

[36] Barison A., Ricci F., Pavon A.G., Muscogiuri G., Bisaccia G., Camastra G., Lazzari M., De Lanzillo C., Raguso M., Monti L. (2023). Cardiovascular Magnetic Resonance in Patients with Cardiac Electronic Devices: Evidence from a Multicenter Study. J. Clin. Med..

[37] Cundari G., Galea N., Rubeis G., De Frustaci A., Cilia F., Mancuso G., Marchitelli L., Catapano F., Carbone I., Catalano C. (2020). Use of the new Lake Louise Criteria improves CMR detection of atypical forms of acute myocarditis. Int. J. Cardiovasc. Imaging.

[38] Dweck M.R., Boon N.A., Newby D.E. (2012). Calcific aortic stenosis: A disease of the valve and the myocardium. J. Am. Coll. Cardiol..

[39] Hein S., Arnon E., Kostin S., Schönburg M., Elsässer A., Polyakova V., Bauer E.P., Klövekorn W.P., Schaper J. (2003). Progression from compensated hypertrophy to failure in the pressure-overloaded human: Heart structural deterioration and compensatory mechanisms. Circulation.

[40] Podlesnikar T., Delgado V., Bax J.J. (2017). Cardiovascular magnetic resonance imaging to assess myocardial fibrosis in valvular heart disease. Int. J. Cardiovasc. Imaging.

[41] Wong T.C., Piehler K., Meier C.G., Testa S.M., Klock A.M., Aneizi A.A., Shakesprere J., Kellman P., Shroff S.G., Schwartzman D.S. (2012). Association between extracellular matrix expansion quantified by cardiovascular magnetic resonance and short-term mortality. Circulation.

[42] Chin C.W.L., Everett R.J., Kwiecinski J., Vesey A.T., Yeung E., Esson G., Jenkins W., Koo M., Mirsadraee S., White A.C. (2016). Myocardial Fibrosis and Cardiac Decompensation in Aortic Stenosis. JACC Cardiovasc. Imaging.

[43] Treibel T.A., Kozor R., Schofield R., Benedetti G., Fontana M., Bhuva A.N., Sheikh A., López B., González A., Manisty C. (2018). Reverse Myocardial Remodeling Following Valve Replacement in Patients With Aortic Stenosis. J. Am. Coll. Cardiol..

[44] Everett R.J., Tastet L., Clavel M.A., Chin C.W.L., Capoulade R., Vassiliou V.S., Kwiecinski J., Gomez M., Beek E.J.R., Van White A.C. (2018). Progression of hypertrophy and myocardial fibrosis in aortic stenosis: A multicenter cardiac magnetic resonance study. Circ. Cardiovasc. Imaging.

[45] Pelliccia A., Sharma S., Gati S., Bäck M., Börjesson M., Caselli S., Collet J.P., Corrado D., Drezner J.A., Halle M. (2021). 2020 ESC Guidelines on sports cardiology and exercise in patients with cardiovascular disease. Eur. Heart J..

[46] D’Ascenzi F., Anselmi F., Piu P., Fiorentini C., Carbone S.F., Volterrani L., Focardi M., Bonifazi M., Mondillo S. (2019). Cardiac Magnetic Resonance Normal Reference Values of Biventricular Size and Function in Male Athlete’s Heart. JACC Cardiovasc. Imaging.

[47] McDiarmid A.K., Swoboda P.P., Erhayiem B., Lancaster R.E., Lyall G.K., Broadbent D.A., Dobson L.E., Musa T.A., Ripley D.P., Garg P. (2016). Athletic Cardiac Adaptation in Males Is a Consequence of Elevated Myocyte Mass. Circ. Cardiovasc. Imaging.

[48] Swoboda P.P., Garg P., Levelt E., Broadbent D.A., Zolfaghari-Nia A., Foley A.J.R., Fent G.J., Chew P.G., Brown L.A., Saunderson C.E. (2019). Regression of Left Ventricular Mass in Athletes Undergoing Complete Detraining Is Mediated by Decrease in Intracellular but Not Extracellular Compartments. Circ. Cardiovasc. Imaging.

[49] Swoboda P.P., McDiarmid A.K., Erhayiem B., Broadbent D.A., Dobson L.E., Garg P., Ferguson C., Page S.P., Greenwood J.P., Plein S. (2016). Assessing Myocardial Extracellular Volume by T1 Mapping to Distinguish Hypertrophic Cardiomyopathy From Athlete’s Heart. J. Am. Coll. Cardiol..

[50] Mordi I., Carrick D., Bezerra H., Tzemos N. (2016). T1 and T2 mapping for early diagnosis of dilated non-ischaemic cardiomyopathy in middle-aged patients and differentiation from normal physiological adaptation. Eur. Heart J. Cardiovasc. Imaging.

[51] Małek Ł.A., Barczuk-Falęcka M., Werys K., Czajkowska A., Mróz A., Witek K., Burrage M., Bakalarski W., Nowicki D., Roik D. (2019). Cardiovascular magnetic resonance with parametric mapping in long-term ultra-marathon runners. Eur. J. Radiol..

[52] Missenard O., Gabaudan C., Astier H., Desmots F., Garnotel E., Massoure P.L. (2021). Absence of cardiac damage induced by long-term intensive endurance exercise training: A cardiac magnetic resonance and exercise echocardiography analysis in masters athletes. Am. J. Prev. Cardiol..

[53] Gaudreault V., Tizon-Marcos H., Poirier P., Pibarot P., Gilbert P., Amyot M., Rodés-Cabau J., Després J.P., Bertrand O., Larose E. (2013). Transient myocardial tissue and function changes during a marathon in less fit marathon runners. Can. J. Cardiol..

[54] Tahir E., Scherz B., Starekova J., Muellerleile K., Fischer R., Schoennagel B., Warncke M., Stehning C., Cavus E., Bohnen S. (2020). Acute impact of an endurance race on cardiac function and biomarkersof myocardial injury in triathletes with and without myocardialfibrosis. Eur. J. Prev. Cardiol..

[55] Gastl M., Achmann V., Christidi A., Janzarik N., Veulemans V., Haberkorn S., Holzbach L., Jacoby C., Schnackenburg B., Berrisch-Rahmel S. (2021). Cardiac magnetic resonance T2 mapping and feature tracking in athlete’s heart and HCM. Eur. Radiol..

